# Does a familial subtype of complex regional pain syndrome exist? Results of a systematic review

**DOI:** 10.1080/24740527.2019.1637249

**Published:** 2019-08-01

**Authors:** S. Modarresi, E. Aref-Eshghi, D. M. Walton, J. C. MacDermid

**Affiliations:** a Health & Rehabilitation Science, Western University, London, Ontario, Canada; b Department of Pathology and Laboratory Medicine, Western University, London, Ontario, Canada; c School of Physical Therapy, Western University, London, Ontario, Canada; d Department of Surgery, Western University, London, Ontario, Canada; e Roth|McFarlane Hand and Upper Limb Centre, St. Joseph’s Health Care, London, Ontario, Canada; f School of Rehabilitation Sciences, McMaster University, Hamilton, Ontario, Canada

**Keywords:** systematic review, complex regional pain syndrome, CRPS, familial

## Abstract

**Background and Objective**: Complex regional pain syndrome (CRPS) is a chronic condition characterized by severe regional pain, allodynia, hyperalgesia, and functional impairment. The aim of this systematic review is to investigate whether a familial subtype of CRPS (fCRPS) exists and to determine whether people with fCRPS have specific characteristics.

**Methods**: Databases CINAHL, Medline, PsycINFO, and PubMed were searched with no date limitation. Quality of reporting was assessed using the Scottish Intercollegiate Guidelines Network scale and the Joanna Briggs Institute’s checklists.

**Results**: Eight studies were included. Family relationships were defined as any immediate (i.e., parents or siblings) or blood relatives. A combination of participants with known or unknown causes for CRPS was recruited. The studies in this review support the potential for the existence of fCRPS, although this included less than 25% of those affected. People with potential fCRPS showed more severe symptoms, more sites involved, a higher percentage of spontaneous onset, and earlier age at onset. An elevated sibling recurrence risk ratio of 5.6 (95% confidence interval [CI], 3.0 to 9.8) was reported for people under 50. None of the studies established a pattern of heritability. Therefore, the most likely explanation for heritability would be a multifactorial model in which cumulative and interactive Gene × Environment effects may be involved.

**Conclusions**: This systematic review supports the potential for the existence of fCRPS; however, all identified studies used uncontrolled case reports, case series, and case–control designs that cannot provide evidence of causation. Further studies are required to reveal the heritability and genetic structure of fCRPS.

## RÉSUMÉ

**Contexte et objectifs**: Le syndrome de douleur régionale complexe (SDRC) est une maladie chronique qui se caractérise par de fortes douleurs régionales, une allodynie, une hyperalgésie et une déficience fonctionnelle. Le but de cette revue systématique était de vérifier s’il existe un sous-type familial de SDRC (SDRCf) et de déterminer si les personnes atteintes de SDRCf présentent des caractéristiques particulières.

**Méthodes**: Des recherches ont été effectuées dans les bases de données CINAHL, Medline, PsychINFO et PubMed, sans limite de date. La qualité des rapports a été évaluée à l’aide de l’échelle du Scottish Intercollegiate Guidelines Network et des listes de vérification de l’Institut Joanna Briggs.

**Résultats**: Huit études ont été incluses. Les relations familiales ont été définies comme toutes les relations immédiates (i.e. parents, frères ou soeurs), ou les parents consanguins. Une combinaison de participants pour lesquels les causes du SDRC étaient connues et inconnues ont été recrutés. Les études ayant fait partie de cette revue vont dans le sens de la possible existence d’un SDRCf, bien que cette constatation touchait moins de 25 % des personnes affectées. Les personnes souffrant d’un possible SDRCf présentaient des symptômes plus graves, un plus grand nombre de régions touchées, un pourcentage plus élevé de déclenchements spontanés et le déclenchement de la maladie à un plus jeune âge. Un rapport relatif de récurrence élevé, se situant à 5,6 (95 % IC, 3,0 à 9,8) chez les frères et soeurs, a été rapporté pour les personnes de moins de 50 ans. Aucune des études n’établissait de modèle d’héritabilité. L’explication la plus plausible pour l’héritabilité serait donc un modèle mutifactoriel dans lequel les effets cumulatifs des gènes et de l’environnement pourraient interagir.

**Conclusions**: Les conclusions de cette revue systémaique vont dans le sens de l’existence possible du SDRCf; toutefois, toutes les études répertoriées ont eu recours à des devis non contrôlés comme des rapports de cas, des séries de cas et des études cas-témoins qui ne peuvent pas prouver le lien de causalité. D’autres études sont nécesaires pour révéler l’héritabilité et la structure génétique du SDRCf.

## Introduction

Complex regional pain syndrome (CRPS) is a painful and disabling syndrome that can affect the upper and/or lower extremities.^1^ CRPS can be categorized into two types: CRPS I occurs spontaneously in the absence of any confirmed injury to the nerves and CRPS II is a type in which there is a known nerve injury.^2^ CRPS I or II occurs more often in women and can happen at any age, although most studies report an average age of onset of about 40.^3–5^ The clinical features of CRPS are diverse and can include severe regional but nondermatomal pain; allodynia; hyperalgesia; changes in skin temperature, texture, or color; and sudomotor and vasomotor dysfunction.^6^ This multifactorial array of symptoms as well as several potential underlying pathophysiological mechanisms give rise to the term “complex” in CRPS. Due in part to this complexity, the incidence of CRPS, which varies by injury, is poorly understood. Two retrospective population-based studies reported an incidence of 5.46 and 26.2 per 100,000 person-years in 1999^5^ and 2007,^7^ respectively. The much higher incidence reported in 2007 could be because of differences in population characteristics such as ethnicity, socioeconomic aspects, or incidence of fractures but is more likely due to differences in case definitions and validation.^7^

One mechanism proposed to explain the genesis of CRPS is genetics, which, if accurate, can mean that a familial subtype of this syndrome exists. Human genetic studies have revealed associations between CRPS and several major histocompatibility complex alleles. These include human leukocyte antigen (HLA)-DR6, HLA-DR13, HLA-DR2, HLA-DQ1, HLA-B62, and HLA-DQ8,^8–12^ as well as a polymorphism in tumor necrosis factor alpha promotor gene.^13^ A report of the involvement of HLA-1 in the spontaneous development of CRPS provides evidence of an interaction between severity of nerve damage and genetic factors in CRPS susceptibility.^14^ Genome-wide expression profiling using the whole blood has shown that HLA-A29.1, matrix metallopeptidase 9, alanyl aminopeptidase, histidine decarboxylase, granulocyte colony-stimulating factor 3 receptor, and signal transducer and activator of transcription 3 genes were highly expressed in those with CRPS compared to healthy controls.^15^ These findings support a genetic component, indicating that hereditary factors might play a role in the susceptibility to CRPS.

A familial vulnerability to CRPS has been reported.^16,17^ However, in addition to the potential genetic susceptibility to CRPS, it is important to recognize the role of the shared family environment of people with CRPS in its development. A systematic review that investigated the influence of stressful life events on the development of CRPS in adults indicated that there is evidence to support that patients with more experienced stressful events have higher chances of developing CRPS.^18^ A more recent study by Wager and colleagues examined this association in children and their results indicated that children with CRPS experienced more stressful events.^19^ It is possible that siblings or relatives in shared familial environments develop CRPS due to experiencing similar stressful events. Therefore, both genetic and environmental factors associated with CRPS development can potentially contribute to a familial subtype of the disease. As preventive, personalized medicine efforts have become a priority in chronic pain research, understanding the inheritance pattern in addition to the family history of CRPS may offer valuable information regarding risk, prognosis, and treatment decisions.

Here, we systematically review the literature and synthesize studies that have reported on the familial occurrence of CRPS. We investigate whether the available data conclude the existence of a familial subtype of CRPS and, using a qualitative synthesis process, we examine whether the patients belonging to this category have specific characteristics distinguishing them from the nonfamilial cases.

## Methods

Prior to commencing this systematic review, the detailed protocol was registered on PROSPERO, registration number CRD42018097359. The Preferred Reporting Items for Systematic Reviews and Meta-Analyses (PRISMA) guideline and checklist were used to plan and report the results of this study.^20^

### Data Sources and Search Strategy

A detailed systematic search of the literature without date filter was conducted on January 7, 2019. Four major electronic databases were searched: CINAHL, MedLine, PsycINFO, and PubMed. The following keywords were used to search the databases: “complex regional pain syndrome” OR “CRPS” OR “causalgia” OR “reflex sympathetic dystrophy” AND “familial” OR “family” OR “sibling” OR “relatives” OR “familial aggregation” OR “twin studies” OR “heredity” OR “hereditary” OR “heritability” OR “genetic” OR “genetics” OR “migration” OR “adoption.” In addition, the reference lists of extracted review articles and relevant articles with a focus on genetics and CRPS were manually searched.

### Study Selection

The following inclusion criteria were used to select the studies for this systematic review:

- Type of participants: At least one of the participant groups were people with CRPS.
- Type of investigation: Studies that fulfilled all or any of the following criteria:
  ● compared the occurrence of CRPS between therelatives of the patients and the general population;
  ● measured the concordance rate for the occurrenceof CRPS among identical and fraternal twins;
  ● described the occurrence of CRPS among relatedpatients.

No restriction was set by age, sex, race, region of pain, duration of symptoms, or the type of study. The study selection process was performed in six stages by two independent reviewers (SM and EA): (1) databases were searched using the search strategy described above; (2) duplicate articles were removed, (3) the bibliography sections of relevant articles were manually searched; (4) titles were screened; (5) abstracts were screened; (6) and full text of articles was screened against the inclusion criteria. When there was uncertainty about the eligibility of an article, a discussion was held and an agreement was achieved by consensus. We did not include conference proceedings, books, dissertations, or unpublished data in this systematic review.

### Data Extraction and Synthesis

Using a pre-established data extraction table, the following information was extracted by SM and cross-checked by EA from each article that met the inclusion criteria: name of the first author, year of publication, country, sample size, mean age (in years) and its standard deviation, percentage of females in the sample, region of pain, whether there was a known cause, diagnostic criteria used to identify cases, proportion of participants having familial CRPS (fCRPS), study design, type of family relationship, and the specific CRPS characteristics.

The studies included in this review have a variety of designs and methodologies, meaning that a meta-analysis of the results was not possible. Hence, the results are presented as a descriptive summary in accordance with the PRISMA guidelines.^20^

### Quality Assessment of Individual Studies

The quality of the studies in this review was assessed independently by two reviewers (SM and EA). For case–control studies, the tool developed by the Scottish Intercollegiate Guidelines Network was used.^21^ This checklist, which consists of two sections, is a simple tool to assess the risk of bias and quality of individual case–control studies, and it is one of the recommended tools by the Agency for Healthcare Research and Quality.^22^ Section 1 examines the internal validity of the study and consists of 11 questions about the selection of participants, assessment methods, confounders, and statistical analysis. Section 2 reviews the overall assessment of the study by rating the methodological quality of the study based on responses to section 1. For case reports and case series we used the Joanna Briggs Institute’s (JBI) checklists, which are specific tools designed explicitly for these types of studies.^23^ These two JBI checklists are widely accepted tools that have been established by the JBI and collaborators and accepted by the JBI scientific committee following extensive peer review.^23^ The JBI checklist for case reports contains eight questions covering areas such as patient’s demographic characteristics, history and timeline, the current clinical condition of the patient, diagnostic tests or assessment methods, treatment procedure(s), postintervention clinical condition, adverse events, as well as takeaway lessons from the study. The JBI checklist for case series contains ten areas including inclusion criteria, identification and measurement of the condition in a standard and reliable way, clear reporting of the clinical information of the participants, reporting of the outcomes or follow-up results of cases, clear reporting of the presenting site(s)/clinic(s), and appropriateness of the statistical analysis.

The interrater agreement of the quality appraisal evaluation was assessed with Cohen’s kappa coefficient using the Statistical Package for the Social Sciences (version 24.0; SPSS, Inc, Chicago, IL), with a value of at least 0.70 considered acceptable.^24^

In this systematic review, although the quality of evidence for each study is informed by the specific critical appraisal tool for that study design, given the differences in study designs and evaluation tools, the overall quality of evidence is based on the more traditional levels of evidence. According to the Oxford rating system, greater confidence is allocated to results-drawn case–control studies, with confidence decreasing as study designs move through case series and case reports.^25^ Therefore, regardless of individual scores (based on their specific evaluation tool), case series and case reports are considered as having lower levels of evidence compared to case–control studies.

## Results

**Figure 1.**
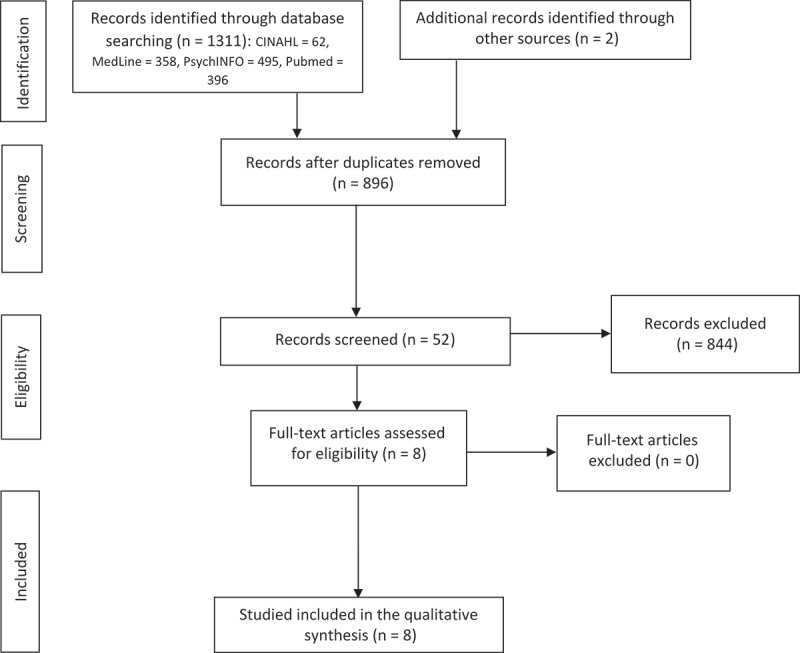
Flow diagram of the study selection process.

In total, 1311 articles were retrieved from the electronic database search (CINAHL = 62, Medline = 358, PsychINFO = 495, PubMed = 396). After removing duplicates and adding articles from the manual search, 896 articles were title screened, of which 844 were excluded because they were clearly not related to the research question. The abstracts and full text of the remaining 52 articles were screened against the inclusion criteria and eight studies were chosen for inclusion. Figure 1 shows a flow diagram of the article selection process in accordance with the PRISMA guideline.^20^

### Study Characteristics

The included studies featured two case reports,^26,27^ three case series,^28–30^ and three case–control studies.^31–33^ The studies in this review represent 1460 people with CRPS, among whom 153 were deemed to be familial cases. Sample sizes ranged from two^27^ to 829,^29^ and the age range of participants was from infancy to 85.^29^ Five of the eight studies included more females than males.^28,29,31–33^ The types of family relationship were any immediate family member, including parents^28,32^ and siblings^26,27,30,31^ or any other blood relative.^29,33^ In the clinical samples, various regions of pain were reported, including hips,^26^ arms,^27–29,31,33^ legs,^28,29,31,33^ or both extremities.^30,31,33^ In one study the cause of CRPS for all patients was unknown,^26^ in two studies the cause was known for all participants,^27,32^ and in the rest a mixture of participants with known or unknown causes for CRPS were recruited; however, it was noted that in these studies the percentage of participants with no cause was less than 25%.^28–31,33^ All five studies that were published after 1994 stated that their method of CRPS diagnosis was based on the criteria endorsed by the International Association for the Study of Pain (IASP) in 1994.^28,30–33^ One study explicitly stated that a medical doctor used these criteria for diagnosis.^31^ One study used the following criteria for CRPS diagnosis:

- Criterion 1: Four out of 5 positive tests from
  - unexplained diffuse pain,
  - difference in skin color relative to the other limb,
  - diffuse oedema,
  - difference in skin temperature relative to the other limb,
  - limited active range of motion.
- Criterion 2: Occurrence or increase of above signs and symptoms after use.
- Criterion 3: Above signs and symptoms present in an area larger than the area of primary injury or operation and including the area distal to the primary injury.^29^

The method for CRPS diagnosis was not reported in two studies.^26,27^ It should be noted that these three studies were published before the official diagnostic criteria for CRPS became available in 1994. Ethics statement was not reported in four studies.^26–29^ A summary of study characteristics is presented in Table 1.

**Table 1.** Summary of study details for papers included in this systematic review (*n* = 8).

Study, country	Sample size	Mean age (SD)	% Female	Region of pain	Known cause?	CRPS diagnostic criteria	% fCRPS	Method of study	Type of family relationship	Quality (%)
Albert and Ott,^26^ Switzerland	3 fCRPS	37 (5.9)	0	Hips	No	n/r	100	Case report	Sibling	87
de Rooji et al,^31^ The Netherlands	405 CRPS (38 fCRPS)	40.6 (15.3)	85	52% arm, 48% leg	13% no cause, 87% trauma, 22% fracture, 27% surgery, 13% soft tissue, 25% other	IASP	9.4	Case–control	Sibling	62
de Rooji et al,^33^ The Netherlands	84 fCRPS	36.7 (14.5)	83	49% arm, 44% leg, 7% both	23% no cause, 77% trauma, 27% fracture, 20% surgery, 20% soft tissue, 9% other	IASP	100	Case–control	Any blood relative	54
Veldman et al.,^29^ The Netherlands	829 CRPS (5 fCRPS)	Varied between 9 and 85; median: 42	CRPS: 76	CRPS: 59% arm, 41% leg	10% no cause, 65% trauma (mostly fracture), 19% surgery, 15% inflammatory process, 4% other (injection, intravenous infusion, cerebrovascular accident)	Specific diagnostic criteria (similar to IASP)	0.6	Case series	Any blood relative	90
Erdmann and Wynn-Jones,^27^ England	2 fCRPS	35.5	50	Both cases: hands	1: injury–slipped 1: injury–vehicle accident	n/r	100	Case report	Sibling	87
Shirani et al,^28^ United States	69 CRPS (9 fCRPS)	fCRPS: 33.8 (12)	fCRPS: 70	77.8% arm, 22.2% leg	22% no cause, 78% trauma or surgery	IASP	13	Case series	Immediate family (parents/siblings)	90
Higashimoto et al.,^30^ United States	8 CRPS	1 at birth; the rest: 6.3 (4.8)	n/r	37.5% leg, 12.5% arm, 37.5% both, 12.5% n/r	28% no cause 2: surgery 1: traumatic fracture 1: a fall	IASP	25; the rest unclear	Case series	2 siblings	90
Huhne et al.,^32^ Germany	60 (12 fCRPS); 21 unaffected relatives	n/r	fCRPS: 92, sCRPS: 71	n/r	100% known cause—traumatic fracture or surgery	IASP	20	Case–control	Immediate family	76

CRPS = complex regional pain syndrome; fCRPS = familial complex regional pain syndrome; n/r = not reported; IASP = International Association for the Study of Pain; sCRPS = sporadic complex regional pain syndrome.

### Quality Assessment Results

The quality assessment showed that all of our studies had an adequately acceptable quality with a mean score of 64% for case–control studies,^31–33^ 87% for case reports,^26,27^ and 90% for all of the case series.^28–30^ When interpreting these scores, it is important to note that they are measured on independent tools specific to the type of study, and these scores cannot be compared with each other across the studies with different designs. Common deficits in reporting among the studies included not using a specific criteria for CRPS diagnosis,^26–28^ not comparing participants and nonparticipants,^31–33^ and not taking confounding factors into account.^31–33^

In this systematic review, it is paramount to recognize that the evidence comes from observational studies, the majority of which are case reports^26,27^ and case series.^28–30^ Thus, even though the quality of individual studies is acceptable, our confidence in the results is not high because of the design of these studies. The interrater agreement between the raters was assessed using the Cohen’s kappa, which showed a value of 0.82 (95% confidence interval [CI], 0.68 to 0.96, *P* < 0.001), corresponding to a substantial level of agreement.^24^

### Incidence of Familial Occurrence of CRPS

A search among studies to identify the rate of fCRPS reveals that indicators of familial aggregation are defined variably and confined to a small portion of the participants. This extent of familial aggregation across studies has been reported to range from negligible^29^ to 25%^30^ of the CRPS population. It should be noted that in the study with negligible reporting of fCRPS (0.6%), the question regarding fCRPS was not consistently asked from all participants,^29^ and we consider this a major methodological limitation. Due to this shortcoming, the incidence of fCRPS in this study may have been underestimated and not reflect the true rate of occurrence. In a study by de Rooij and colleagues, which examined the risk of CRPS in 405 cases, the rate of fCRPS was reported to be 4% in “confirmed” cases.^31^ When including “possibly affected” cases in that same study, the incidence of fCRPS occurrence increased to 6%.^31^ In this study, confirmed cases were those for which a clinician had made a formal diagnosis, but information regarding the possibly affected cases was obtained through self-report by the siblings. In another study of 60 people with CRPS with a history of injury prior to the disease onset, 12 cases (20%) were found to also have affected family members.^32^ A clinical review of an additional 69 cases of CRPS identified a total of four families with more than two members (13%) affected by the condition.^28^ This rate increased to 15% when both confirmed and unconfirmed cases were considered. In this study, the “unconfirmed” cases were not formally examined but the family history was only reported by the patients.^28^ The authors of this study failed to confidently assign a Mendelian pattern of inheritance to the pedigrees and proposed that the fCRPS occurrence might be modified by genetic heterogeneity, variable penetrance, epigenetic regulations, or environmental factors.^28^

### Characteristics of Familial CRPS

The earliest study in this systematic review was published in 1983, reporting the first evidence for a familial form of CRPS.^26^ This case report presented three brothers with sudden occurrences of pain in the hip without any previous injury or trauma. The occurrence of CRPS in hips is rare; however, the authors argue that there was no evidence of other diseases, including cardiac, endocrine, pulmonary, or neurological. The clinical presentations, radiological findings, or course of progression and improvement of the disease was reported to follow the common patterns seen in patients with CRPS; however, the occurrence in a familial form led the authors to propose that a genetic predisposition could be involved.^26^ Of interest, all three brothers had an identical HLA formula (A 1–30 or 31; B 8–37; BW 4–6; DR 7-x; MT 3–1, 2), which is also a rare coincidence.^26^ No article on the familial incidence of CRPS is found again until 1993 when Erdmann and Wynn-Jones described the cases of two siblings presenting with CRPS four years apart.^27^ Both events happened within six weeks following mild injuries to the upper extremities and progressed to vascular pathology, osteoporosis, and eventually distal gangrene. The two cases shared striking similarities in the initiation, progression, and outcome of the disease; both had an initial encouraging response to treatment with the later deterioration of the symptoms leading to distal limb amputations but, interestingly, neither of the siblings suffered from phantom limb pain. In addition, they shared some common psychological factors that are known to be associated with poor prognosis, including lack of motivation and suspected self-interference, ranging from the tight squeezing of the arm through to ligature bruising.^27^ These features indicated that a shared family history might be involved in both the incidence and prognosis of CRPS, but whether the common psychological factor was causal or consequential was not addressed.^27^ Two other studies reported psychological disturbances but only in two of their participants with fCRPS, and they were less severe, including stress,^30^ emotional irritability, and anxiety.^28^ Similarly, it is not clear from these studies whether the psychological disturbances are symptoms of CRPS or they are shared consequences of the severe symptoms. de Rooij and colleagues reported on 31 families with two to five affected family members (84 people with fCRPS), comparing those against cases with no obvious familial connection.^33^ Those with familial links had a younger age at onset, a higher percentage of spontaneous onset, more temperature and color asymmetry, more sweating and trophic disturbances, and more often had multiple affected extremities and dystonia.^33^ A study by Shirani and colleagues also reported that those with a familial connection qualitatively developed the disease at an earlier age and had more migraine headaches and more bilateral involvement compared to the nonfamilial cases.^28^ Another study hypothesized that a subset of pediatric cases of CRPS that also presented with additional neuromuscular conditions might be caused by mitochondrial DNA defects.^30^ Their investigation of 500 patients with CRPS identified seven families with such functional features, mostly gastrointestinal dysmotility, migraine, cyclic vomiting, and chronic fatigue. All of these families met the criteria for a maternal mitochondrial inheritance.^30^ This finding suggested that mitochondrial inheritance might explain some familial cases of CRPS that also present with additional functional symptoms.^30^

### The Magnitude of Familial Involvement in CRPS

The study by de Rooij et al. is the only one to date to have reported on familial aggregation of CRPS.^31^ The analysis of 405 patients with CRPS using sibling recurrence risk ratio in all “possibly affected” siblings was estimated to be 1.8 (95% CI, 1.1 to 2.7), meaning that there is a 1.8-fold increased risk of CRPS occurrence among siblings of affected persons as compared to the general population. When all possibly affected siblings were stratified into age groups, the risk ratio for people under the age of 50 was estimated at 5.6 (95% CI, 3.0 to 9.8), indicating that the risk is much higher in younger persons. The analysis of this cohort for people older than 50 revealed that the risk was not significantly different from the general population (0.6; 95% CI, 0.3 to 1.0). Further detailed evaluation identified 16 confirmed cases in their siblings, which did not indicate a significant aggregation when compared with the incidence of the disease in the general population. Similarly, again restricting the analysis to cases younger than 50 years old, a recurrence risk ratio of 3.4 (95% CI, 1.5 to 6.8) was found, indicating a more pronounced role for hereditary factors in the cases with a younger age of onset.^31^

## Discussion

This systematic review examined the limited pool of evidence on familial occurrences of CRPS to elucidate the extent of risk given family history and whether differences in phenotypes might characterize a familial subtype of CRPS. Given the dearth of evidence, we did not exclude by study design because the available literature is mainly composed of individual case reports, case series, and identification of familial cases among populations affected by CRPS. Only one article specifically studied the familial aggregation in CRPS.^31^ Though the evidence is limited, it does point to the potential for a familial form of CRPS, which accounts for a minority of those affected (i.e., <25%). Less frequent history of trauma and more associated symptoms, diffuse symptoms, and a larger component of central and systemic symptoms may characterize this phenotype. These include more migraine headaches,^28,30^ more temperature and color asymmetry,^33^ more sweating and trophic disturbances,^33^ vascular pathology,^27^ osteoporosis,^27^ distal gangrene,^27^ gastrointestinal motility,^30^ cyclic vomiting,^30^ chronic fatigue,^30^ dystonia,^33^ more sites involved,^33^ bilateral involvement,^28^ a higher percentage of spontaneous onset,^33^ and earlier age at onset,^28,31,33^ though in every case these have been qualitatively explored without inferential analyses. Despite some consistency, the study designs preclude inference and offer little evidence of causation. As such, the current state of evidence can best be summarized as presenting potentially testable hypotheses in more rigorous designs.

One criterion for causation is biologic plausibility,^34^ which the current evidence has started to provide, but the mechanisms underlying familial aggregation are still far from clear. Mitochondrial involvement has been observed in a subset of cases presenting with neuromuscular symptoms in addition to the typical CRPS features,^30^ which may represent an understudied mechanism of this condition. No study was able to establish a pattern of inheritance for the familial group according to Mendel’s laws; thus, the most likely explanation for heritability would be a polygenic or multifactorial model, although other forms of non-Mendelian genetic involvement, such as Gene × Environment and gene–gene interactions, and epigenetics cannot be ruled out. For instance, it has been found that people living in disadvantaged neighborhoods have a higher chance of developing chronic pain after motor vehicle collisions; of interest, this effect is modifiable by a single nucleotide polymorphism in the promoter of FKBP5, a functional regulator of glucocorticoid receptor sensitivity.^35^ In addition, epigenetic modifications including DNA methylation and histone modifications are proposed to be involved in the establishment of gene regulatory status in primary sensory neurons of dorsal root ganglion associated with pain hypersensitivity in chronic pain conditions.^36^ Epigenetic markers are modifiable, and at least one study has shown that downregulating phosphorylation of serine 10 (S10) in histone 3 in superficial spinal dorsal horn neurons reduces hyperalgesia and provides a promising new direction for chronic pain therapy.^37^ Shared environments by siblings and relatives should also be accounted for as a plausible mechanism for the occurrence of fCRPS. Previous research has shown that adverse life events are associated with the development of CRPS,^18,19^ and there is a high chance for the possibility of family members to share adverse life events. In fact, the most significant stressful life events that have been shown to be associated with the development of CRPS are family related.^19^ Sherry and Weisman examined the social environment of children with CRPS and indicated that CRPS can be a stress-related disease because their participants experienced stressful events such as marital discord between parents and sexual abuse.^38^ These results were further confirmed in a study by Kachko and colleagues that reported that migration history, low socioeconomic status, divorced parents, chronic disorders of other family members, and controlling behavior of parents were seen in patients with CRPS.^39^

Due to the clinical heterogeneity of CRPS and its rarity, genome-wide association studies have been difficult to design, and the only genome-wide association study performed to date has failed to identify a common single nucleotide polymorphism to be associated with the disease.^40^ The only genetic associations reported for CRPS are with HLA genotypes,^11,14,41^ which were not reproduced in a subsequent study,^40^ possibly due to phenotype heterogeneity, which may have weakened the association signals. All of these studies have been conducted on small sample sizes. Therefore, large-scale genetic association studies aimed at detecting slight genetic variations associated with CRPS are still needed to identify genetic variants contributing to the heritability of CRPS. Given that our systematic review indicates that hereditary factors may have a more prominent role in a subset of patients, performing such studies on familial cases has the potential to improve power in detecting such small genetic variant associations.

The major limitation of this systematic review rests on the limitation of the existing literature in that there are no publications attempting to answer the question regarding the involvement of genetics versus the environment in fCRPS. A lack of consistent case definitions, inconsistent collection of family history, retrospective data (subject to recall bias), poor integration of clinical and genetic phenotype test protocols, and the lack of large cohorts needed to estimate rare events with precision have all undermined the confidence in the findings to date. Familial aggregation measures should not be confused with evidence for causation by genetics. At best, they may reveal a potential role for shared characteristics between family members, composed of both genetics and environment. To distinguish between the involvement of genetics versus environment, the heritability of CRPS should be estimated using indices most commonly measured using twin studies. According to our search, no such twin studies have been conducted, and doing so would be challenging if not impossible in humans, in that both twins would also need to have been exposed to the same or similar inciting event (e.g., trauma). This may be more readily conducted using animal models. A second limitation is that the current literature is lacking in data from participants stratified by phenotype. Phenotypic heterogeneity in CRPS is an important indicator that is not accounted for in estimating the familial recurrence risk ratio of CRPS. In addition, the only aggregation study performed to date on CRPS will need to be replicated in a different cohort. This is of particular importance given that both familial aggregation and heritability can be population specific.^42^ Another limitation is that all of the studies used uncontrolled case reports, case series, and case–control designs that cannot provide evidence of causation. However, they provide evidence of association, which can construct a basis for investigation of the causative factors. Finally, three of our included studies were published before the IASP diagnostic criteria for CRPS became available in 1994; therefore, the diagnostic accuracy of these studies may be lower. It is essential to state that the diagnosis (and treatment) of CRPS has been a challenge to clinicians for a long time, often leading to false diagnoses even following the 1994 release of IASP official diagnostic criteria.^43^ Our inclusion of results from papers published prior to these criteria may add heterogeneity to the results, but we felt that it is important, given the dearth of literature, to conduct a comprehensive review that highlights the gaps in current knowledge. Despite the limitations in the pool of evidence, prior authors have identified the potential for a familial subtype of CRPS and, given that the presentation and prognosis for this familial type seems to be particularly negative, this systematic review does have the potential to impact clinical decisions. In our opinion, awareness of this potential is a valuable contribution of this article. We have provided potential mechanistic explanations that appear to be promising directions for further exploration in this relatively understudied clinical condition.

In summary, the findings of this review indicate the potential for a familial risk for CRPS to exist, particularly in those with an earlier age at onset and more severe presentation. Establishment of a familial subtype of CRPS justifies estimating the role of environment versus genetics in the disease and conducting molecular studies and searching for predisposing genes. This will require substantial improvements in standardized data collection and the use of other study designs, such as twin and genetic association studies. Such studies might provide a clearer understanding of the pathophysiology of the disease and help targeted screening and therapy for patients at risk.
